# The Treatment of Oesophageal Perforation after Anterior Cervical Spinal Surgery

**DOI:** 10.1155/2019/2350958

**Published:** 2019-04-01

**Authors:** Li-Sheng Hu, Zhen-Quan Wu, Li-Min Zou, Yong-Can Huang

**Affiliations:** ^1^Shenzhen Key Laboratory of Spine Surgery, Department of Spine Surgery, Peking University Shenzhen Hospital, Shenzhen, China; ^2^Department of Orthopaedics, Yunfu People's Hospital, Yunfu, China; ^3^Shenzhen Engineering Laboratory of Orthopaedic Regenerative Technologies, Orthopaedic Research Center, Peking University Shenzhen Hospital, Shenzhen, China

## Abstract

Oesophageal perforation is a rare complication occurring during or after cervical spine surgery, and the risk factors are not well understood. This study presents a case of a 25-year-old man with oesophageal perforation after anterior cervical spine surgery. It is suggested that four factors (anatomical structure, mechanism of trauma, implant dislodgment, and the operation) could induce postoperative oesophageal perforation after cervical spine surgery performed using the anterior surgical approach. A comprehensive understanding and early management of this complication are necessary for successful therapy.

## 1. Introduction

Oesophageal perforation is one of the devastating complications of cervical spine surgery performed using an anterior surgical approach which has been reported sporadically over the past few decades [1, 2]. The underlying risk factors of this complication are far from well understood. Hence, in this study, we carefully summarise the treatment of a patient who suffered an oesophageal perforation after cervical spinal surgery. We then review the updated literature and discuss the possible risk factors of oesophageal injuries.

## 2. Case Report

**Figure 1:**
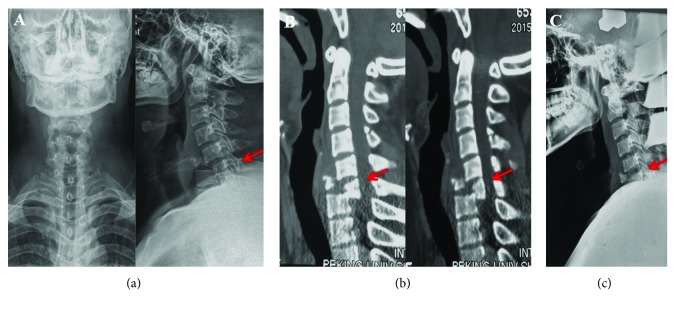
(a) Cervical spine X-ray. (b) Cervical spine CT. (c) X-ray under skull traction.

**Figure 2:**
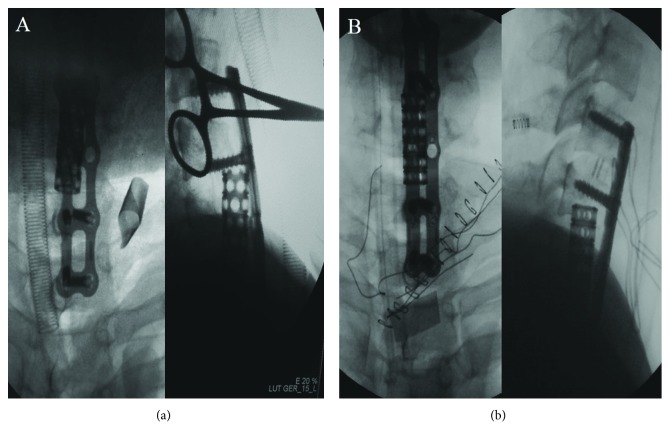
(a) X-ray during the first surgery. (b) X-ray during the second surgery.

**Figure 3:**
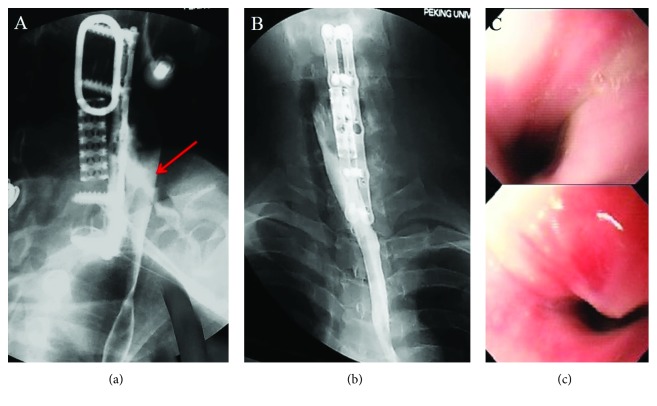
(a) Gastrografin swallow study. (b) Gastrografin swallow study 8 weeks later. (c) UGI endoscopy.

A 25-year-old male patient with a C6 fracture and dislocation (AO classification C1.2.4) was treated with skull traction following an unsuccessful manual reduction on the day of the accident (Figure 1). After six-day preoperative preparation and traction, he had cervical spinal surgery for a C6 corpectomy, a C4/5-C6/T1 discectomy, and fusion of the C-spine using a titanium mesh cage. Radiographs indicated that the mesh cage was not well positioned postoperatively (Figure 2(a)). The patient was thus taken for secondary surgery to revise the plate and mesh cage (Figure 2(b)). A white purulent discharge from the surgical site was observed 30 days after the first operation. An oesophageal fistula at the level of C6 was confirmed by a gastrografin swallow test and laryngoscopy (Figure 3(a)). The patient was immediately taken for a thorough wound debridement; subsequently, continuous extensive irrigation was performed, intravenous vancomycin was started, and gastric decompression was done using continuous nasogastric tube drainage. Four weeks later, the results of three continuous cultures of bacteria were negative. After eight weeks, an upper GI endoscopy and a repeat gastrografin swallow were performed, and the irrigation and nasogastric tubes were removed. The patient reported no discomfort at three-month follow-up (Figures 3(b) and 3(c)).

## 3. Discussion

Oesophageal perforation is a relatively rare but destructive event which leads to life-threatening consequences such as mediastinitis, septicaemia, and meningitis [3]. Oesophageal perforation can be divided into early (symptoms occurring within 1 week of surgery) and delayed (discomfort occurring weeks or even years after surgery) presentations [3–5]. The early variety could be attributable to an iatrogenic injury such as instrumental retraction, damage from the original injury, or inappropriate fixation and fusion; the latter type was invariably due to the dislodgement of the implants [6]. This patient from the case discussed herein belongs to the second type of iatrogenic injury. The pathophysiological mechanism of an oesophageal perforation/fistula is still unclear [3]. The anatomical structure at the anterior cervical spine may have resulted in an oesophageal perforation for the following reasons.

First, the oesophageal wall at the C6 level was a relatively weak part of the oesophagus; at the Lannier triangle, the posterior oesophageal mucosa is extremely thin, as it is only covered by one layer of fascia [7, 8]. In this case, such characterisation might explain the presence of the fistula.

Second, other researchers have suggested that the etiology, such as a C-spine fracture caused by hyperextension force, was more likely to induce a visible or invisible oesophageal injury compared to other etiologies, such as disc herniation, metastatic diseases, and deformity [9–11], because the sharp bony fragments would stab directly into the surrounding soft tissue. This patient, who had a hyperextensive injury to the cervical spine, might have subsequently had an invisible tear from the bony fragment during the trauma. Such force would not only compress the posterior vertebra but also stretch the soft tissue anteriorly and reduce the oesophagus resistance to the stretching force from the retractor during surgery.

Third, the dislodgment of bony grafts or implants might cause subsequent oesophageal complications after surgery [10, 12, 13]. Our patient had a dislodgement of the titanium mesh cage at the early period after the first cervical spine surgery; postoperative radiographs showed that loosening the cage oblique ventrally and directly led to compression on the anterior soft tissue. Immediate replacement of the plate and cage was performed to prevent the continuous compression on the oesophagus; notably, no visible tear was found during this reexploration. Soft tissue damage during the revision was inevitable, which meant the oesophageal perforation would occur more easily compared to a normal oesophagus.

Therefore, we confirm that multilevel surgery, prolonged duration, direct injury from the surgical instruments, traction during the surgery, and invasive endotracheal intubation are the underlying risk factors for oesophageal perforation [14–16].
